# Rapid and efficient generation of mature retinal organoids derived from human pluripotent stem cells via optimized pharmacological modulation of Sonic hedgehog, activin A, and retinoic acid signal transduction

**DOI:** 10.1371/journal.pone.0308743

**Published:** 2024-08-09

**Authors:** Tokiyoshi Matsushita, Akishi Onishi, Takahiro Matsuyama, Tomohiro Masuda, Yoko Ogino, Masaaki Kageyama, Masayo Takahashi, Fumiaki Uchiumi

**Affiliations:** 1 Faculty of Pharmaceutical Sciences, Department of Gene Regulation, Tokyo University of Science, Noda, Chiba, Japan; 2 Product Discovery, Ophthalmology Innovation Center, Santen Pharmaceutical Co., Ltd., Ikoma, Nara, Japan; 3 Laboratory for Retinal Regeneration, RIKEN Center for Biosystems Dynamics Research, Kobe, Hyogo, Japan; 4 Cell and Gene Therapy in Ophthalmology Laboratory, Baton Zone Program, RIKEN, Wako, Saitama, Japan; Affiliated Hospital of Jiangsu University, CHINA

## Abstract

Human retinal organoids have become indispensable tools for retinal disease modeling and drug screening. Despite its versatile applications, the long timeframe for their differentiation and maturation limits the throughput of such research. Here, we successfully shortened this timeframe by accelerating human retinal organoid development using unique pharmacological approaches. Our method comprised three key steps: 1) a modified self-formed ectodermal autonomous multizone (SEAM) method, including dual SMAD inhibition and bone morphogenetic protein 4 treatment, for initial neural retinal induction; 2) the concurrent use of a Sonic hedgehog agonist SAG, activin A, and all-*trans* retinoic acid for rapid retinal cell specification; and 3) switching to SAG treatment alone for robust retinal maturation and lamination. The generated retinal organoids preserved typical morphological features of mature retinal organoids, including hair-like surface structures and well-organized outer layers. These features were substantiated by the spatial immunostaining patterns of several retinal cell markers, including rhodopsin and L/M opsin expression in the outermost layer, which was accompanied by reduced ectopic cone photoreceptor generation. Importantly, our method required only 90 days for retinal organoid maturation, which is approximately two-thirds the time necessary for other conventional methods. These results indicate that thoroughly optimized pharmacological interventions play a pivotal role in rapid and precise photoreceptor development during human retinal organoid differentiation and maturation. Thus, our present method may expedite human retinal organoid research, eventually contributing to the development of better treatment options for various degenerative retinal diseases.

## Introduction

Retinal organoids are self-organized retinal tissues derived from pluripotent stem cells (PSCs), including induced PSCs (iPSCs), which recapitulate some physiological and pathophysiological features of retinal development and diseases. Since their initial establishment, much effort has been focused on investigating the molecular mechanisms of various retinal diseases using retinal organoids [1–3]. For example, Mayerl et al. [4] reported that unlike interphotoreceptor matrix proteoglycan 2 (IMPG2) knockout mice, retinal organoids generated using retinitis pigmentosa patient-derived iPSCs displayed typical clinical disease phenotypes, including photoreceptor outer segment loss, when IMPG2 mutations were inserted. More importantly, the authors found that CRISPR/Cas9-based genomic correction of these mutations remarkably ameliorated the observed disease phenotypes. Likewise, QR-110, an antisense oligonucleotide designed as a novel therapy for Leber congenital amaurosis type 10, has entered the clinical development stage based on the finding that it restored centrosomal protein of 290 kDa protein function in patient-derived retinal organoids with its gene mutations [5]. Thus, retinal organoids have been established as indispensable research tools that are alternative or complementary to animal disease models.

Various methods for differentiating human PSCs into retinal organoids have been developed and improved over the past decade [6–11]. Under specific culture conditions, these PSCs form embryoid bodies that subsequently differentiate into optic vesicles and eventually laminate retinal organoids resembling retinal development in the body [6–11]. Based on the morphological characterization of retinal organoids in bright-field images, Capowski et al. [12] proposed three stages of retinal maturation. 1) In the first stage, designated stage 1, retinal organoids appear as a small enclosed sphere-like structure with a thick phase-bright outer layer and a thin phase-dark core that comprises neural retinal progenitors and differentiated retinal ganglion cells (RGCs). 2) During stage 2, organoids further grow and differentiate, resulting in an enlarged sphere with a thinner outer layer and thicker dark core, where RGCs disappear. Then, retinal photoreceptor precursors, horizontal cells, and amacrine cells are generated. 3) Stage 3 organoids acquire a hair-like structure on the surface of the clearly organized outer layer that represents a more mature outer retinal structure, including mature photoreceptors with inner/outer segment-like structures and Müller glia. The authors also proposed that stage 3 retinal organoids are optimal for disease modeling when photoreceptor structures and functions are damaged. However, it is generally recognized that such a complex maturation process of retinal organoids requires 120–170 days after isolation of the neural retinal cluster for a floating culture, which corresponds to 150–200 days following the initiation of PSC differentiation, regardless of the differentiation methods used for organoid generation [12]. Therefore, accelerated maturation of retinal organoids may provide significant benefits to investigators who strive to expedite their studies, particularly in the field of drug discovery and development for the treatment of photoreceptor degenerative diseases.

One of the major factors that can accelerate retinal organoid generation is the combined and timed activation of multiple signaling pathways governing retinal cell fate, as suggested by fibroblast growth factor signaling [13]. In fact, Zerti et al. [14] found that various pharmacological agents, such as all-*trans* retinoic acid (RA) and triiodothyronine, promote photoreceptor maturation in human retinal organoids, depending on the timing of their treatment alone or in combinations. Similarly, another group successfully improved the efficiency of human photoreceptor precursor generation following the application of COCO, a multifunctional antagonist of the Wnt, transforming growth factor beta, and bone morphogenetic protein (BMP) signaling pathways [15]. Although these studies provided methods to promote efficient photoreceptor differentiation in human retinal organoids, they did not accelerate the differentiation process. To date, the most promising finding was that the replacement of all-*trans* RA with 9-*cis* RA led to increased rhodopsin expression as a sign of retinal maturation on day 120 following the initiation of differentiation of human PSCs [16]. This method reduces the timeframe required for rod photoreceptor differentiation and maturation, but not for cones [16]. Thus, we hypothesized that the more precise titration of culture conditions, particularly pharmacological treatments throughout the differentiation procedure, could drastically improve and accelerate the photoreceptor maturation process in human retinal organoids.

In this study, we used human iPSC-derived retinal organoids to optimize three critical culture conditions for pharmacological interventions: 1) selection of differentiation-promoting agents, 2) individual use or their combinations, and 3) timing of their application. We found that our newly developed and optimized photoreceptor differentiation method accelerated human retinal organoid maturation resembling stage 3 organoids within 90 days, which is two-thirds the timeframe necessary for traditional methods.

## Materials and methods

### Human iPSC culture maintenance

This study was conducted in compliance with the principles of the Declaration of Helsinki. All experimental procedures were reviewed and approved by the institutional ethics committee of Santen Pharmaceutical Co., Ltd. (Osaka, Japan) for the use of human materials. The human iPSC lines 1231A3 and M8 were established and provided by the Center for iPS Cell Research and Application (CiRA), Kyoto University (Kyoto, Japan) [17] and RIKEN (Wako, Japan) [18], respectively. Both cell lines were maintained in StemFit medium (Ajinomoto Co., Inc., Tokyo, Japan) on laminin 511-E8 fragment-coated plates (Nippi, Inc., Tokyo, Japan), according to CiRA’s instructions. To initiate iPSC differentiation, the cells were seeded in a 6-well plate at a density of 5,000 cells/well and cultured for 10 days in StemFit medium.

### Induction of neural retinal progenitors in two-dimensional culture

When the cells formed tightly packed colonies 10 days after seeding, the culture medium was switched to differentiation medium containing 10% KnockOut Serum Replacement (Thermo Fisher Scientific, Waltham, MA, USA), 0.1 mM nonessential amino acids (Thermo Fisher Scientific), 1 mM sodium pyruvate, 100 U/mL penicillin, 100 mg/mL streptomycin, and 450 μM 1-monothioglycerol (Sigma-Aldrich, St. Louis, MO, USA) in Glasgow’s Minimum Essential Medium (Thermo Fisher Scientific). SMAD signaling inhibitors, SB431542 (10 μM; FUJIFILM Wako Pure Chemical Co., Tokyo, Japan) and LDN193189 hydrochloride (100 nM; Sigma-Aldrich), were added to the culture media at differentiation day (DD) 0 and DD1, and then replaced with 3 nM BMP4 (R&D Systems, Minneapolis, MN, USA) alone from DD1 to DD3 to direct the PSCs toward the neuroectoderm and retinal fate.

### Retinal organoid differentiation in floating culture from DD10 to DD90

**Fig 1:**
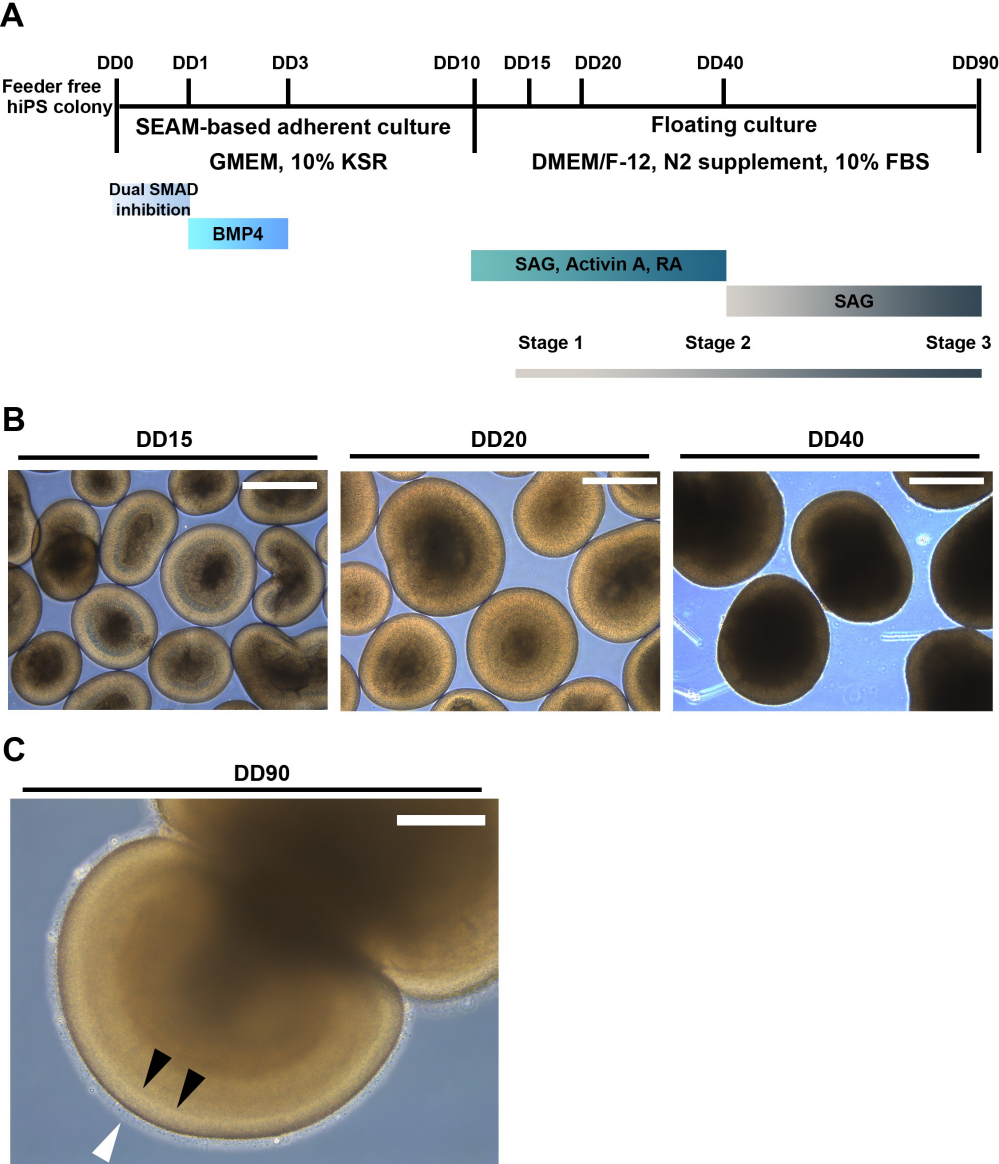
Schematic diagram describing a novel method for human retinal organoid generation and representative images of their morphological features. (A) Culture conditions and the timing of treatment with differentiation-promoting agents are indicated. The time after the initiation of the differentiation of retinal organoids differentiation was indicated as the DD. The timing and combinations of pharmacological treatments were carefully designed to accelerate retinal organoid differentiation and maturation. (B) Representative phase contrast images of retinal organoids differentiated at DD15, DD20, and DD40. Scale bar: 500 μm. (C) Higher magnification of a phase contrast image of retinal organoids fully differentiated at DD90. White and black arrowheads indicate the hair-like structure and the definitive border (outer nuclear layer/outer plexiform layer border), respectively. Scale bar: 200 μm. The representative images were arbitrarily chosen from eight independent experiments.

The tightly packed clusters of neural retinal progenitors were gently lifted by scraping and transferring to a floating culture in maturation medium containing DMEM/F-12, GlutaMAX supplement (Thermo Fisher Scientific), 10% fetal bovine serum (Biosera Inc., Marikina, Philippines), N2 supplement (Thermo Fisher Scientific), and 100 μM taurine (FUJIFILM Wako Pure Chemical Co.) at DD10. The medium was changed every other day over the course of the floating culture. A Sonic hedgehog (SHH) signal/smoothened agonist (SAG, 100 nM), 100 ng/mL activin A, 1 μM all-*trans* RA (all from FUJIFILM Wako Pure Chemical Co.), or their combinations were added to the maturation medium from DD10 to DD40. SAG was continuously present in the maturation medium throughout the rest of the culture period (Fig 1A). Under light microscopy, retinal organoids differentiated by the method represented the defined outer lamina with budding of hair-like structures around DD90 (Fig 1C), and the differentiation rate was calculated based on the presence of the structural characteristics of the stage 3 retinal organoid at DD90.

### Immunostaining

**Fig 2:**
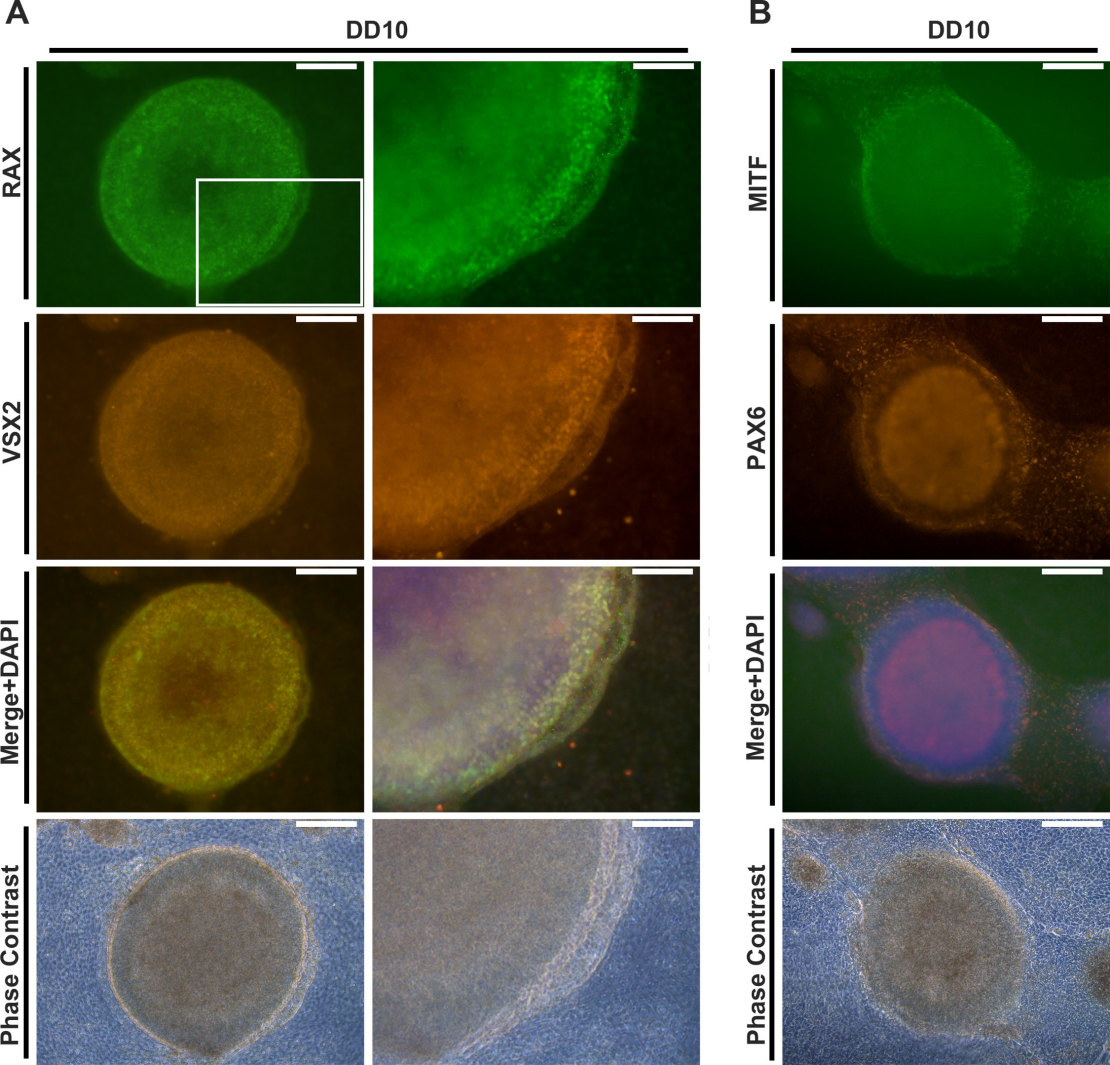
Neural retinal progenitor differentiation in the modified SEAM method. Human iPSC colonies were cultured using the SEAM method with specific pharmacological treatments. These colonies were initially exposed to dual SMAD inhibition and then switched to BMP4 treatment. (A) RAX (green)- and VSX2 (red)-double-positive neural retinal progenitor cells emerged at DD10, as revealed by immunocytochemistry of developing retinal clusters. Scale bars: 200 μm (left) and 100 μm (right). (B) MITF- (green) and PAX6-positive (red) retinal progenitor/RPE precursors. Scale bar: 200 μm. Nuclei were stained with DAPI (blue). Differentiating cells were examined in six wells in three independent experiments. Representative images were chosen arbitrarily.

**Fig 3:**
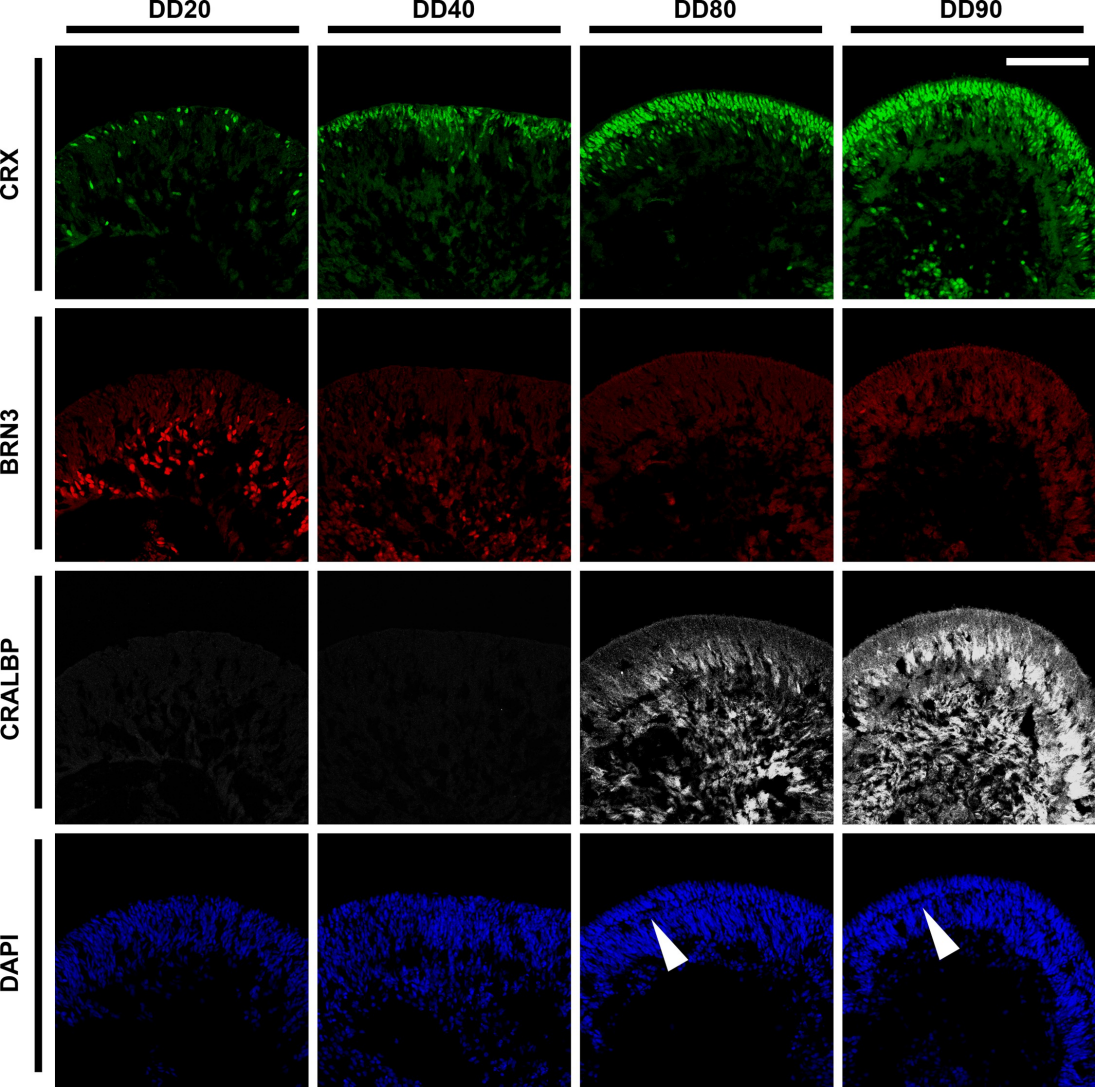
Accelerated differentiation of human retinal organoids into photoreceptors, RGCs, and Müller glia in floating culture with optimized pharmacological treatments. Human retinal organoids were generated in the presence of three differentiation-promoting agents (SAG, activin A, and RA) and harvested at each DD throughout the 90-day study period. Retinal organoids were cryosectioned and immunostained with antibodies against various retinal cell marker proteins. CRX (green): Photoreceptor precursor/photoreceptor, BRN3 (red): RGC, CRALBP (white): Müller glia. White arrowheads indicate the intermediate layer between two consecutive nuclear layers, presumably representing the OPL-like structure. Nuclei (blue) were stained with DAPI. Scale bar: 100 μm. Ten sections prepared from ten retinal organoids were examined in three independent experiments. Representative images were chosen arbitrarily.

**Fig 4:**
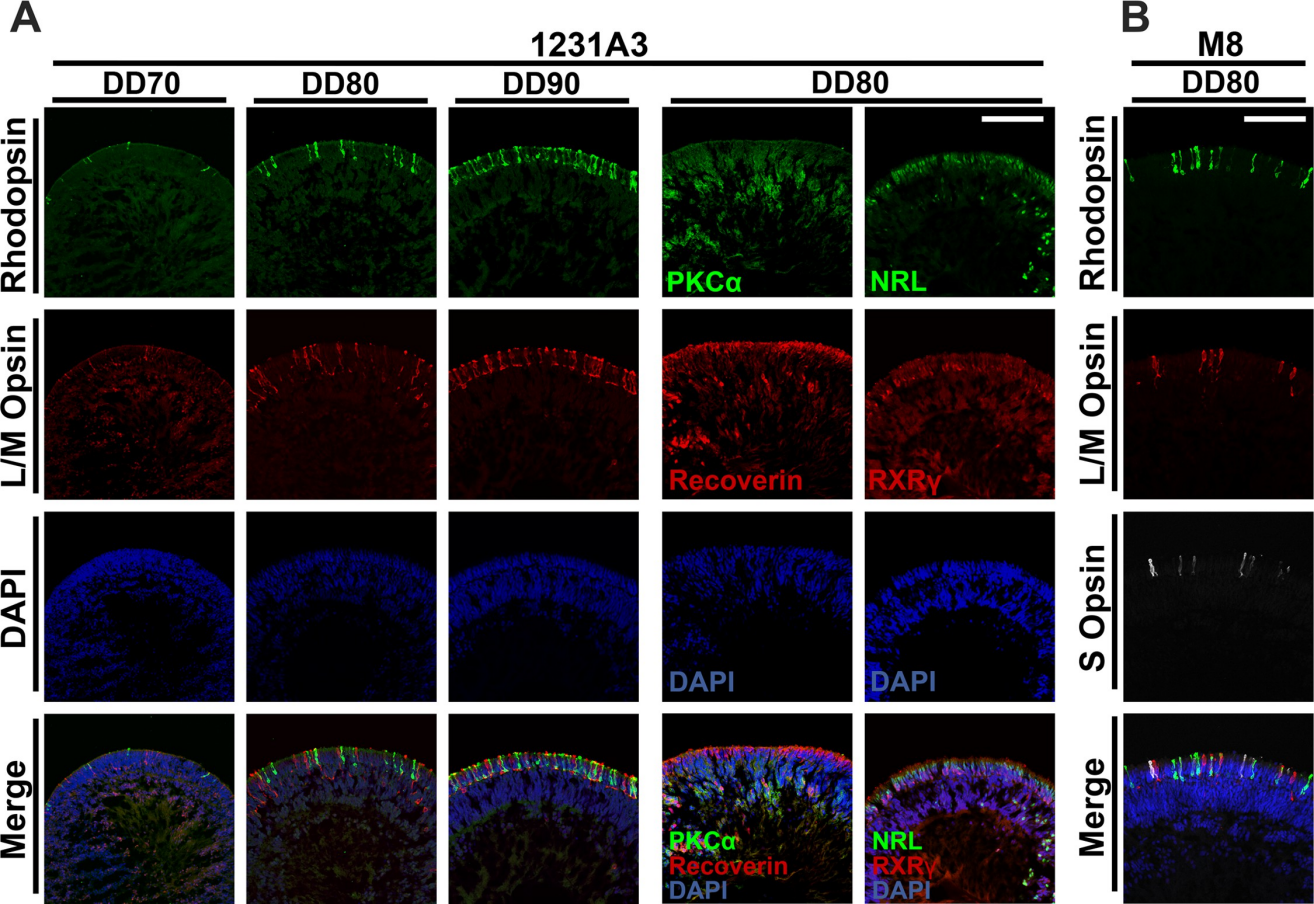
Rapid maturation of photoreceptors and bipolar cells in human retinal organoids derived from two different iPSC lines. (A) Retinal organoids derived from the human iPSC line 1231A3 at a late DD were cryosectioned and immunostained with antibodies against various retinal cell marker proteins. DD70 to DD90: Rhodopsin (green) and L/M opsin (red) for mature rod and cone photoreceptors, respectively; DD80: PKCα (green, bipolar cell marker), NRL (green, rod photoreceptor marker), recoverin (red, photoreceptor marker), and RXRγ (red, cone photoreceptor marker). Scale bar: 100 μm. (B) Mature photoreceptors in retinal organoids derived from the human iPSC line M8. rhodopsin (green), L/M opsin (red), and S opsin (white, cone photoreceptor marker) at DD80. Scale bar: 100 μm. Nuclei (blue) were stained with DAPI for all experiments. Ten sections prepared from ten retinal organoids were examined in three independent experiments. Representative images were arbitrarily chosen from three independent experiments.

**Fig 5:**
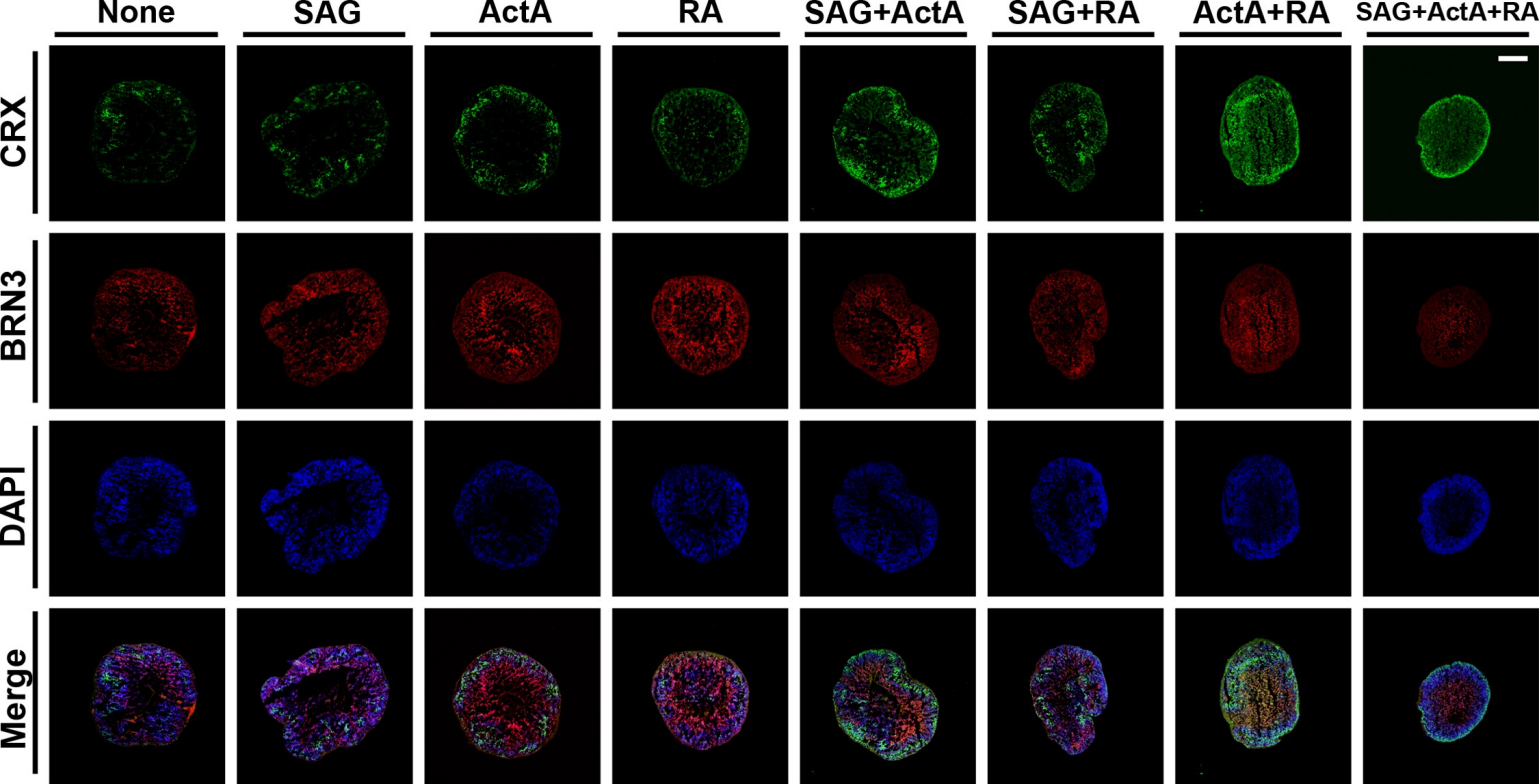
Promoting effects of concurrent pharmacological modulation of multiple signal transduction on photoreceptor differentiation in the outermost layer of human retinal organoids. Retinal organoids were cultured in maturation medium supplemented with SAG, activin A, and RA, alone or in combination from DD10 through DD40. At DD40, retinal organoids were cryosectioned and immunostained with antibodies against the photoreceptor precursor/photoreceptor marker CRX (green) and RGC marker BRN3 (red). Nuclei (blue) were stained with DAPI for all experiments. Scale bar: 100 μm. Ten sections prepared from ten retinal organoids were examined in three independent experiments. Representative images were chosen arbitrarily.

**Fig 6:**
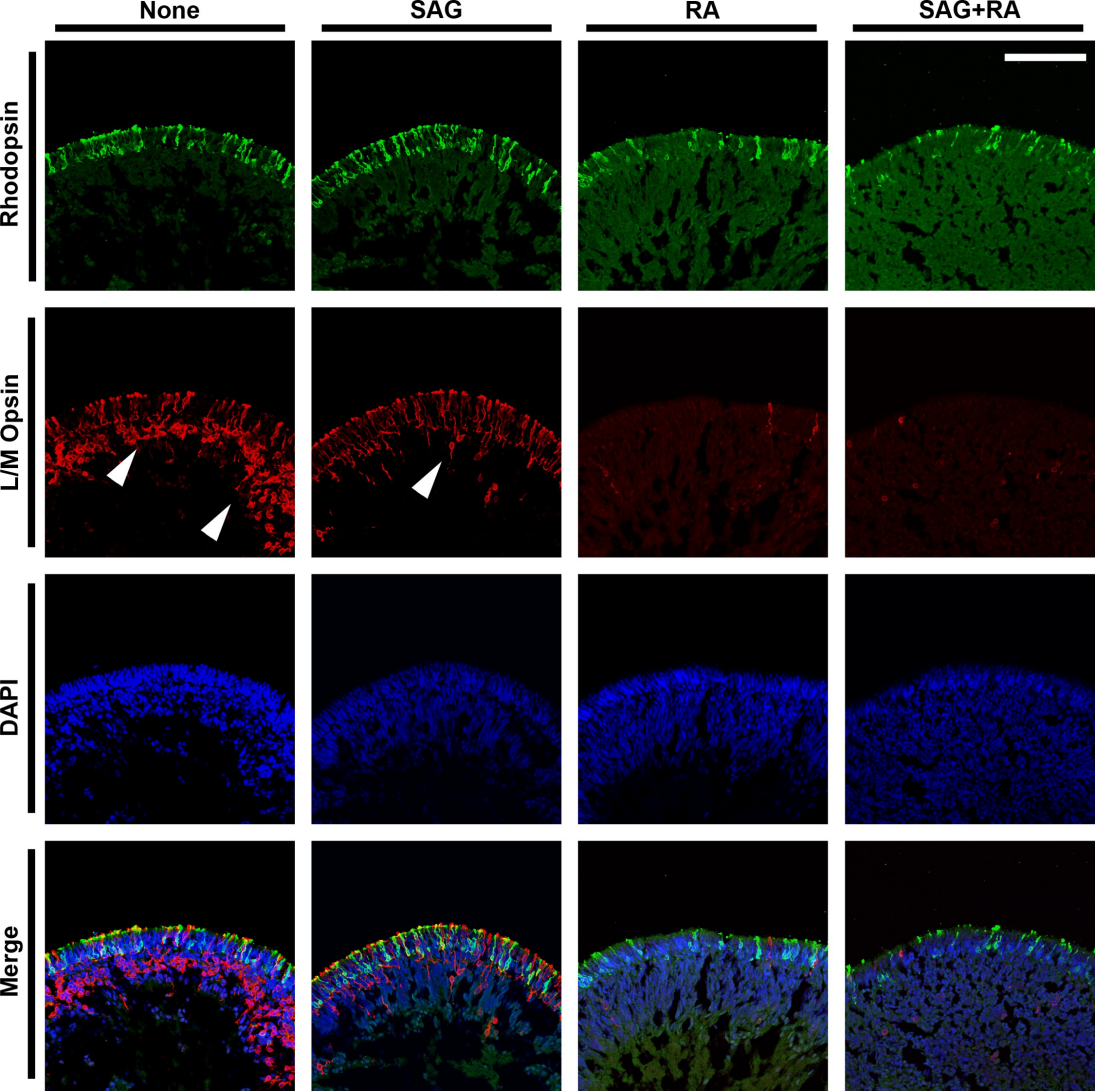
Determinant effect of SAG on mature photoreceptor lamination in human retinal organoids. Retinal organoids were cultured in maturation medium supplemented with SAG and RA, alone or in combination from DD40 through DD90. At DD90, retinal organoids were cryosectioned and immunostained with antibodies against the photoreceptor marker rhodopsin (green) and L/M opsin (red). White arrowheads indicate ectopic cone photoreceptors that developed in the inner region of retinal organoids. Nuclei (blue) were stained with DAPI for all the experiments. Scale bar: 100 μm. Ten sections prepared from ten retinal organoids were examined in three independent experiments. Representative images were chosen arbitrarily.

Cells in the adherent culture and retinal organoids were harvested on the indicated days of differentiation. Cells and retinal organoids were fixed in 4% paraformaldehyde at 4°C for 30 min. For immunohistochemistry, the fixed retinal organoids were immersed in 7.5% sucrose/phosphate-buffered saline (PBS) and subsequently in 30% sucrose/PBS prior to cryopreservation. Then, the fixed retinal organoids were embedded in O.C.T. compound (Sakura Fineteck Japan Co., Ltd, Tokyo, Japan) and frozen at –80°C. The embedded organoids were cryosectioned using a Leica CM3050S cryostat (Leica Biosystems, Nußloch, Germany) at 10 μm thickness. For immunostaining, the fixed cells and cryosections were blocked in 5% heat-inactivated horse serum (Thermo Fisher Scientific) and 0.05% Triton X-100 in PBS at room temperature for 30 min, followed by incubation with a primary antibody at 4°C overnight. The next day, the slides were washed with PBS and incubated with secondary antibody at room temperature for 2 h. Nuclei were counterstained with 4′,6-diamidino-2-phenylindole (DAPI; Thermo Fisher Scientific). The stained sections were mounted using the PermaFluor Aqueous Mounting Medium (Thermo Fisher Scientific). Fluorescent microscopic images (Figs 2–6) were acquired using DMi8 inverted fluorescence microscope (Leica Microsystems, Wetzlar, Germany) and FX3000 confocal laser scanning microscope (Olympus, Tokyo, Japan). More detailed information regarding the antibodies used in this study is provided in the Supporting Information (S1 Table).

### Quantitative polymerase chain reaction

Retinal organoids treated with SAG, activin A, RA, or their combinations were harvested at DD20 and subjected to quantitative PCR (qPCR). Total RNA was purified from retinal organoids using NucleoSpin RNA (Macherey-Nagel GmbH & Company KG, Düren, Germany), according to the manufacturer’s instructions. Then, total RNA was reverse-transcribed using the PrimeScript RT Reagent Kit (Takara Bio Inc., Shiga, Japan), and qPCR was performed with the TB Green Premix Ex Taq II (Takara Bio) using the QuantStudio 3 Real-Time PCR System (Thermo Fisher Scientific). The primer sequences used for qPCR are listed in the Supporting Information (S2 Table). The gene expression levels of each gene were normalized to those of *GAPDH* and represented as fold change from the baseline expression level obtained at DD0 in human iPSCs. Statistical analyses were performed using GraphPad Prism version 10.1.0 for Windows (GraphPad Software, La Jolla, CA, USA), with three biological replicates for each experimental group. Tukey’s multiple comparison test was originally performed followed by Dunnett’s multiple comparison test, as indicated in the figure legend, to compare the concurrent use of the three agents group to the other treatment groups.

## Results

Fig 1A and 1B summarize the experimental procedures established for accelerated human retinal organoid differentiation and maturation, and their morphological features at each stage, respectively. Initially, tightly packed human iPSC colonies were subjected to dual SMAD inhibition from DD0 to DD1, and then switched to treatment with BMP4 from DD1 to DD3, resulting in differentiation of iPSCs into neural retinal progenitors at DD10. Subsequently, these colonies were placed in a floating culture and exposed to three differentiation-promoting agents: SAG, activin A, and RA. At approximately DD15, neural retinal progenitors exhibited morphological features of stage 1 retinal organoids, which included a continuous phase-bright thick outer layer and a phase-dark thin core. The retinal organoids further differentiated and acquired stage 2-like morphological features with an enlarged phase-dark core while losing the phase-bright outer layer on DD40. Following the withdrawal of activin A and RA at DD40, SAG alone further promoted the maturation of retinal organoids as hair-like surface structures along with clearly organized outer layers at DD90 (Fig 1C). These observations are consistent with the morphological features of stage 3 retinal organoids [12]. The differentiation of human iPSC lines 1231A3 and M8 by the method presented in Fig 1 was confirmed in eight and three individual experiments, respectively. The mean rates of successful induction of stage 3 retinal organoids at DD90 from the human iPSC lines 1231A3 and M8 were 60.9 ± 9.9% and 49.3 ± 6.1% (mean ± standard deviation), respectively.

To characterize the human retinal organoids generated by the above-established method further, immunostaining was performed to examine the expression of various retinal cell-specific markers at each stage of the retinal organoids (Fig 2). For the initial differentiation of retinal organoids, we used the self-formed ectodermal autonomous multizone method [19,20], which efficiently directs human iPSCs toward the ectoderm and retinal fate. We also modified this method to include both the dual SMAD inhibition protocol [21] and BMP4 treatment [10] to accelerate retinal progenitor differentiation further. As shown in Fig 2A, dual SMAD inhibition and subsequent BMP4 treatment induced retina and anterior neural fold homeobox protein 2 (RAX) and visual system homeobox 2 (VSX2) double-positive neural retinal progenitors at DD10 in tightly packed clusters. These clusters of neural retinal progenitors were surrounded by microphthalmia-associated transcription factor (MITF) and paired box 6 (PAX6) double-positive cells developed at the outer shell and in its surrounding area, which are presumably retinal pigment epithelium (RPE) precursors (Fig 2B). Because these two layers were clearly separated, only RAX/VSX2 double-positive clusters were gently scraped and further differentiated in a floating culture containing SAG, activin A, and RA followed by SAG treatment alone. Remarkably, cone-rod homeobox (CRX), a transcription factor expressed in photoreceptors and their precursors, sporadically emerged as early as DD20, and its expression levels gradually increased during the rest of the culture period (Fig 3, top panel). More importantly, these CRX-positive cells were localized along the outermost layer in stage 2 retinal organoids at DD40 and then preferentially organized as a well-defined outer layer structure in stage 3 retinal organoids at DD90. Unlike CRX expression, expression of the RGC marker brain-specific homeobox/POU domain protein 3 (BRN3) was initially detected in stage 1 retinal organoids at DD20, but diminished and disappeared thereafter, accompanied by the increased expression of the Müller glial marker protein cellular retinaldehyde-binding protein (CRALBP) (Fig 3, middle). Notably, nuclear staining of DD80 and DD90 retinal organoids showed a clear separation between the outer nuclear layer (ONL) and the inner nuclear zone, indicating the development of an outer plexiform layer (OPL)-like structure in the retinal organoids (Fig 3, bottom). The rod and cone photoreceptor markers, rhodopsin and L/M opsin protein, respectively, were detected as early as DD70, and well-aligned along the outermost layer at DD80 and DD90 (Figs 4A and S1). At DD80, these expression patterns were similar to those of other rod (neural retina leucine zipper [NRL] and Recoverin) and cone photoreceptor markers (retinoid X receptor gamma [RXRγ] and recoverin) accompanied by expression of the rod bipolar cell marker protein kinase C alpha (PKCα) (Fig 4A), indicating mature stage 3 retinal organoids. To confirm these results, we used another human iPSC line M8, demonstrating a similar differentiation pattern of retinal organoids at DD80, accompanied by the exclusive expression of rhodopsin, L/M opsin, and S opsin along the outermost layer (Fig 4B). Taken together, our established method can generate stage 3 retinal organoids within 90 days following the initiation of iPSC differentiation, as evident from the well-defined outer retinal structure, including rod and cone photoreceptors and other cell populations, such as bipolar cells, horizontal cells, amacrine cells and Müller glia (Figs 4 and S2).

**Fig 7:**
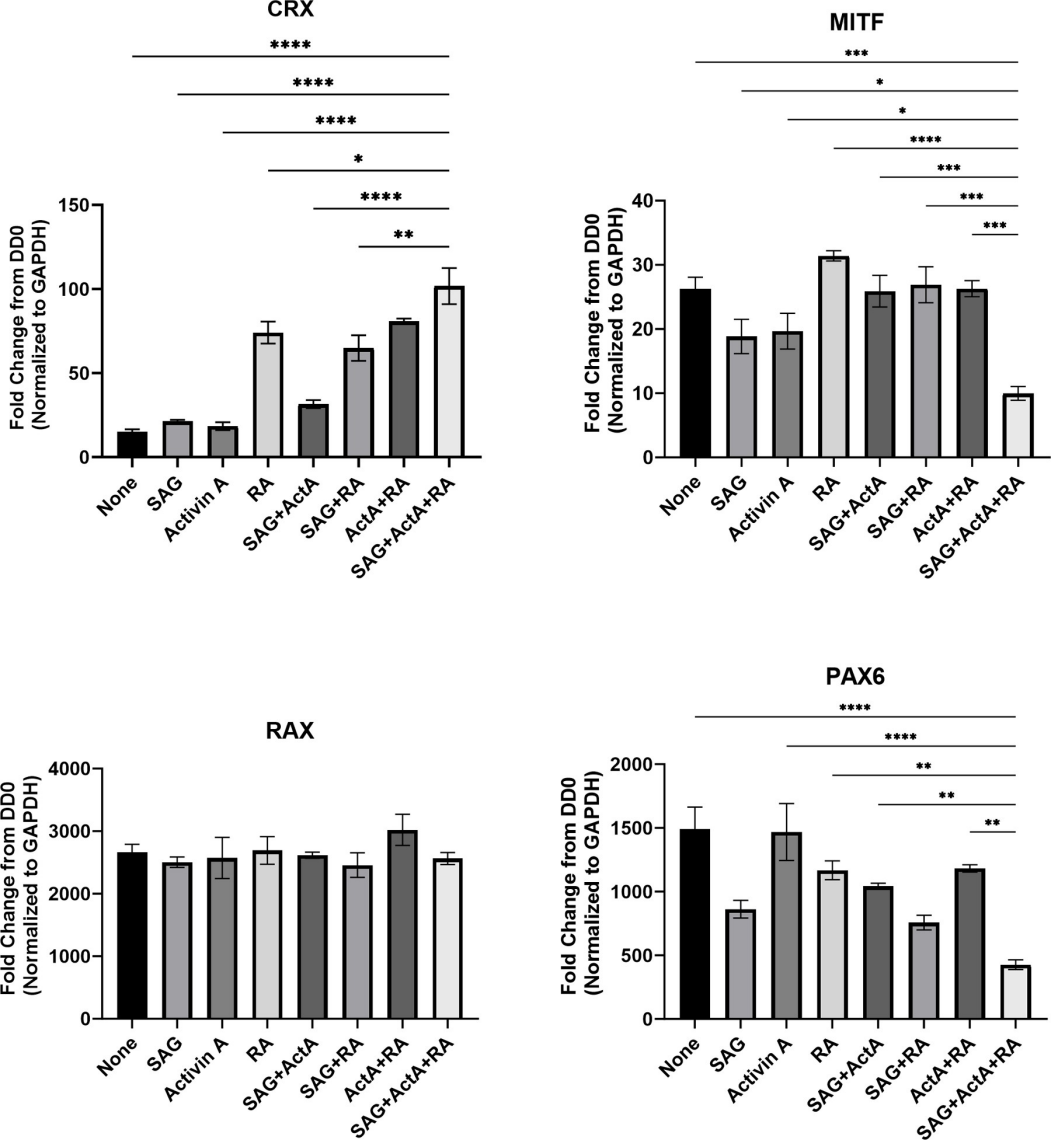
Altered gene expression of various retinal progenitor cell makers in human retinal organoids treated with differentiation-promoting agents. Retinal organoids were cultured in maturation medium supplemented with SAG, activin A, and RA, alone or in combination, from DD10 through DD20. Retinal organoids were harvested and subjected to gene expression analysis for *CRX*, *MITF*, *RAX*, and *PAX6* by qPCR. Each expression level was normalized to that of *GAPDH* and is indicated as a fold-change from the respective basal expression levels observed at DD0. Data are representative from three independent experiments with three biological replicates in each treatment group. Error bars represent standard error of the mean. *P < 0.05, **P < 0.01, ***P < 0.001, ****P < 0.0001 by Dunnett’s multiple comparison test.

To determine the impact of pharmacological interventions on retinal organoid differentiation, we examined the effects of SAG, activin A, and RA alone, or in combination on neural retinal progenitor differentiation at stages 1 and 2 (Figs 5 and 7) and their maturation at stage 3 (Fig 6). The addition of SAG, RA alone, or their combination to the maturation medium from DD10 to DD40 had no major effect on CRX expression patterns compared with the maturation medium alone as seen at DD40 (Fig 5). By contrast, activin A alone slightly increased CRX expression levels, and this effect was enhanced when combined with the other agents. More importantly, the concurrent use of SAG, activin A, and RA altered the spatial distribution patterns of CRX, resulting in the localization of CRX-positive cells in the outermost layer of retinal organoids. This change was accompanied by minimal BRN3 expression levels. Surprisingly, RA alone, but not activin A or SAG, significantly upregulated *CRX* gene expression at DD20 (Fig 7), although it did not alter the protein expression at DD40. This effect of RA further increased in combination with the other two agents. As expected, the increased *CRX* gene expression was associated with reduced expression of the progenitor maker genes, *MITF* and *PAX6*, following the concurrent use of the three agents, although *RAX* gene expression did not change under any treatment condition.

Finally, we investigated the effects of RA and SAG treatment, alone or in combination, on stage 3 retinal organoid maturation. At this stage, activin A was eliminated from the culture medium at DD40 for all treatment conditions because activin A reportedly converts the neural retina to RPE precursors [22]. As shown in Fig 6, SAG treatment alone preserved the laminated rhodopsin and L/M opsin expression in the outermost layer at DD90. However, RA treatment alone or in combination with SAG diminished rhodopsin expression and even eliminated L/M opsin expression. Interestingly, while preserving rhodopsin and L/M opsin expression, SAG treatment alone dramatically suppressed the displaced photoreceptor generation around the inner part of retinal organoids (Figs 6 and S3). Thus, concurrent treatment with the three differentiation-promoting agents followed by SAG treatment alone appears to be the most critical component of our established method for accelerating retinal organoid differentiation and maturation.

## Discussion

In the present study, we successfully established a novel method for accelerated human retinal organoid generation, resulting in a significantly reduced timeframe required for the generation of mature stage 3 retinal organoids as defined in previous studies [12,19]. Our method required only 90 days for stage 3 retinal organoid generation, whereas other methods, including the 9-*cis* RA-based method, require at least 120 days [15,19]. The major differences between our method and others are: 1) a modified SEAM method, including dual SMAD inhibition and BMP4 treatment, to initiate neural retinal induction; 2) the concurrent use of SAG, activin A, and RA to accelerate retinal cell differentiation; and 3) switching to SAG treatment alone for further maturation and lamination of photoreceptors in retinal organoids.

One may argue that accelerated retinal organoid generation is simply due to the genuine differentiation property of the human iPSC line used in this study. The human iPSC line 1231A3 established and distributed by Kyoto University was used as the standard human iPSC line [17]. Kuwahara et al. [20] demonstrated that this cell line possesses the ability to generate retinal organoids, but requires the same amount of time for retinal organoid maturation as that observed in other conventional methods. More importantly, we demonstrated using another cell line, M8, established by RIKEN [18] that our method could generate mature retinal organoids in the same amount of time. Retinal organoids generated by our method using these two different cell lines were morphologically similar to stage 3 organoids, and their spatial retinal marker protein expression patterns were indistinguishable from those in retinal organoids generated by any other conventional method, for instance SFEBq method [20] (S4 Fig). Applicability of the presented protocol for the broad range of pluripotent stem cell lines has to be further demonstrated as a future study as well as a detailed characterization of cellular identity in retinal organoids using single-cell RNA sequencing [23,24]. At present, we cannot deny the possibility that they may be functionally different and exhibit unique disease phenotypes when retinal disease-causing mutations are incorporated into their genomes. Further studies are underway to address this possibility using human retinal organoids generated using our method and other methods.

For initial neural retinal induction, we modified the SEAM method that was originally developed for corneal epithelial cell differentiation by Hayashi et al. [25]; later, the method was applied for retinal organoid generation by Li et al. [26] because this method efficiently directs pluripotent cells toward ectodermal lineages, including the neuroectoderm and retina. Interestingly, Chambers et al. [21] demonstrated that combined treatment with two different SMAD signaling inhibitors, Noggin and SB431542, promotes the rapid neural conversion of human embryonic SCs (ESCs). Another group tested several signaling modulators to differentiate human ESCs into retinal cells and identified BMP4 as the most effective modulator among all of the tested pharmacological interventions [10]. Therefore, we adapted and optimized these pharmacological treatments by using the SEAM method for rapid neural retinal induction. In fact, we found that initial dual SMAD inhibition and subsequent BMP treatment effectively accelerated the retinal specification of iPSCs. This modified method requires only 10 days to generate neural retinal progenitors, whereas other conventional methods require 3 to 4 weeks. Thus, our modified SEAM method contributes significantly to the rapid and efficient generation of primitive human retinal organoids.

The most crucial component of our method for the accelerated maturation of human retinal organoids is the optimized pharmacological treatments during the culture period from DD10 to DD40. In particular, the concurrent use of three differentiation-promoting agents, SAG, activin A, and RA, seems to navigate retinal progenitors to photoreceptor precursor cells. This notion was substantiated by the findings that concurrent treatment with the three agents led to preferentially laminated CRX expression in the outermost layer of retinal organoids at DD40. Consistently, in the presence of these agents, the *CRX* gene was upregulated and accompanied by reduced *MITF* and *PAX6* gene expression, which regulate other retinal cell lineages [27,28]. Unexpectedly, RA treatment alone or in combination with another agent also increased expression of the *CRX* gene without altering its protein expression, suggesting the complex transcriptional and posttranscriptional regulation of CRX expression in response to the concurrent use of the three agents [29,30]. With the concurrent use of the three agents, BRN3-positive RGCs were observed in DD20 retinal organoids, but the cells almost disappeared in DD40 retinal organoids, suggesting accelerated RGC differentiation and cell death due to the loss of axonal connection, as suggested in a previous study [8]. Thus, it is most likely that concurrent treatment with the three agents plays a significant role in the accelerated differentiation of human retinal organoids to generate CRX-positive photoreceptor precursors aligned along the outermost layer of retinal organoids. To our knowledge, this is the first report highlighting the importance of concurrent treatment with an SHH agonist, activin A, and RA to accomplish the rapid generation of well-defined outer retinal structures in human retinal organoids.

The other key component of our method is to switch the pharmacological treatment from concurrent use of the three agents to SAG alone in the culture period from DD40 to DD90. Notably, SAG treatment alone led to a more aligned outer retinal structure representing the ONL, which maintained rhodopsin and L/M opsin expression in the outermost layer of DD90 retinal organoids while suppressing ectopic L/M opsin expression in the inner portion. These patterns were accompanied by the increased expression of other retinal cell markers, namely, CRALBP in Müller glial cells, PKCα in rod bipolar cells, PROX1 in horizontal cells, calretinin in amacrine cells, and recoverin, RXRγ, and NRL in photoreceptors. These results suggest that SAG alone is indispensable and sufficient for retinal organoid maturation during the culture period. Unlike SAG, RA treatment alone or in combination with SAG generated a more disorganized outer retinal layer with diminished rhodopsin expression and minimal L/M opsin expression. This finding is consistent with a previous report that RA delays the initiation of photoreceptor gene expression in retinal organoids, although RA is generally regarded as a promoting factor for rod photoreceptor differentiation [31,32]. Taken together, we successfully optimized the culture conditions to accelerate retinal organoid maturation with a special focus on pharmacological interventions, including combinations of differentiation-promoting factors and the timing of their treatment. Our optimized method may significantly contribute to the accelerated and improved quality of retinal organoid maturation, where a well-organized outer retinal structure is developed and ectopic photoreceptor generation is suppressed.

Although this study was not designed to elucidate the exact molecular mechanisms underlying accelerated retinal organoid maturation, the complex crosstalk among SHH, activin A, and RA signaling in determining retinal cell fate may explain how the combination and timing of pharmacological interventions accelerated retinal organoid differentiation. SHH, activin A, and RA signaling individually promote retinal development and photoreceptor differentiation [32–36]. Interestingly, Zhang et al. [37] studied the direct reprogramming of human fibroblasts into RPE-like cells, demonstrating that simultaneous activation of SHH and RA signaling dramatically increased CRX expression, although it was mainly associated with the gene expression signature characterizing the RPE lineage, including MITF and PAX6. Activin A treatment in the presence of vitamin A, which is presumably converted to RA, promotes postmitotic photoreceptor precursor differentiation from mouse ESCs with increased CRX expression, but no changes in other photoreceptor genes including rhodopsin [38]. By contrast, our study demonstrated that all three signaling pathways were activated simultaneously during stages 1 and 2 of retinal organoid differentiation, and only SHH signaling was required for further maturation into stage 3 retinal organoids expressing mature photoreceptor marker genes and proteins. Thus, these three pathways may individually and cooperatively contribute to the recruitment and activation of transcription factors required for each stage of retinal organoid differentiation and maturation. Further studies are underway to determine the precise molecular mechanisms underlying the retinal organoid development accelerated by our optimized pharmacological interventions.

In summary, we established a novel method to accelerate human retinal organoid differentiation and maturation under optimized culture conditions. The key components of these culture conditions comprise unique pharmacological approaches: 1) a modified SEAM method with consecutive treatment with dual SMAD inhibitors and BMP4 treatment; 2) concurrent treatment with SAG, activin A, and RA; and 3) switching to SAG treatment alone. We demonstrated that our method required only 90 days to generate stage 3 human retinal organoids with well-organized outer retinal structures. Interestingly, our method also improved the quality of retinal organoids by suppressing ectopic photoreceptor generation following the treatment of stage 2 retinal organoids with SAG alone. These improved features of retinal organoid generation may benefit investigators who need high-quality retinal organoids for disease modeling, including drug discovery and development for the treatment of retinal diseases.

## Conclusions

The present study provides a novel and improved method using optimized pharmacological interventions for accelerated differentiation and maturation of human retinal organoids. Given the significantly reduced timeframe for retinal organoid generation, we believe that our method may expedite translational research from the bench to the clinic, particularly in the field of drug discovery and development, including the identification and optimization of therapeutic candidates.

## Supporting information

S1 Table Antibodies used in the study. (DOCX)

S2 Table Primers for qPCR. (DOCX)

S1 Fig Rapid generation of photoreceptors in retinal organoids differentiated by the present method from DD70 toward DD90. Retinal organoids differentiated from the human iPSC line 1231A3 by the present method were harvested at DD70, DD80, and DD90. Retinal organoids differentiated from the human iPSC line M8 by the present method were harvested at DD80. Retinal organoids were cryosectioned and immunostained with antibodies against photoreceptor markers rhodopsin, L/M opsin, and S opsin. A 300 μm-wide region of interest was randomly selected from 18 sections prepared from 18 retinal organoids, and fluorescent microscopic images were acquired using an FX3000 confocal laser scanning microscope (Olympus, Tokyo, Japan). Cell numbers were manually counted using FIJI-ImageJ software (National Institute of Health, Bethesda, USA). Data represent an arbitrarily chosen experiment from three independent experiments. Each bar represents the mean standard error of the photoreceptor numbers obtained from 18 individual sections of the 18 retinal organoids. ****P < 0.0001 by Tukey’s multiple comparison test. Statistical analyses were performed using GraphPad Prism version 10.1.0 for Windows (GraphPad Software, La Jolla, CA, USA).
(TIF)

S2 Fig Expression of retinal cell markers in DD90 retinal organoids differentiated by the present method. Retinal organoids differentiated from the human iPSC line 1231A3 by the present method were harvested at DD90. Retinal organoids were cryosectioned and immunostained using antibodies against various retinal cell marker proteins. PROX1 (upper, green): horizontal cells, calretinin (upper, red): amacrine cells, S opsin (lower, green): short-wave cone photoreceptors. The nuclei (blue) were stained with DAPI. Scale bar: 100 μm. The experiments were performed thrice with three different differentiation lots, in which six sections from six organoids were examined. Representative images were chosen arbitrarily.
(TIF)

S3 Fig Effects of SAG and RA supplementation from DD40 to DD90 on photoreceptor generation and localization in DD90 human retinal organoids. Retinal organoids differentiated from human iPSC 1231A3 were cultured in maturation medium supplemented with SAG and RA, either alone or in combination, from DD40 to DD90. At DD90, retinal organoids were cryosectioned and immunostained with antibodies against the photoreceptor markers rhodopsin, L/M opsin, and S opsin. A 300 μm-wide region of interest was randomly selected from 10 sections prepared from 10 retinal organoids, and fluorescent microscopic images were acquired using an FX3000 confocal laser scanning microscope (Olympus, Tokyo, Japan). Cell numbers were manually counted using Fiji-ImageJ software (National Institute of Health, Bethesda, USA). Data represent an arbitrarily chosen experiment from three independent experiments. Each bar represents the mean standard error of the photoreceptor numbers obtained from 10 individual sections. (A) The effect of SAG and RA, either alone or in combination, on photoreceptor generation in ONL. **P < 0.01, ****P < 0.0001 by Dunnett’s multiple comparison test. (B) The effect of SAG on suppressing the ectopic expression of L/M opsin was analyzed by comparing the sub-tissue localization of L/M opsin-positive cells. ***P < 0.001 by Student’s t-test. Statistical analyses were performed using GraphPad Prism version 10.1.0 for Windows (GraphPad Software, La Jolla, CA, USA).
(TIF)

S4 Fig Expression of retinal cell markers in DD120 and DD222 retinal organoids differentiated from the human iPSC line M8 cells by a conventional method. Retinal organoids were differentiated from human iPSC M8 according to the SFEBq method reported by Kuwahara et al. [20] and harvested at DD120 and DD222. Retinal organoids were cryosectioned and immunostained using antibodies against photoreceptor markers. The nuclei (blue) were stained with DAPI. (A) DD120 retinal organoids differentiated by the SFEBq method showed few rhodopsin-positive cells and no L/M opsin-positive cells, while immature rod and cone photoreceptor markers, NRL and RXRγ, respectively, were expressed. Scale bars: 500 μm (left) and 100 μm (middle and right). (B) DD222 retinal organoids differentiated by the SFEBq method showed rhodopsin (green) and L/M opsin (red) expression. Scale bar: 50 μm. The experiments were performed thrice with three different differentiation lots, in which six sections from six organoids were examined. Representative images were chosen arbitrarily.
(TIF)
