# Supplementary material for: Rapid and efficient generation of mature retinal organoids derived from human pluripotent stem cells via optimized pharmacological modulation of Sonic hedgehog, activin A, and retinoic acid signal transduction
Source: PLoS One. 2024 Aug 9;19(8):e0308743. doi: 10.1371/journal.pone.0308743 (PMC11315325; doi:10.1371/journal.pone.0308743)
Supplement: S1 Table — (DOCX) [file pone.0308743.s001.docx]

**S1 Table. Antibodies used in the study**

| **Antibody product name** | **Dilution** | **Catalog No.** | **Manufacturer** |
| --- | --- | --- | --- |
| Rabbit anti-Mouse Rx | 1:1,000 | M228 | Takara Bio Inc. |
| Sheep anti-Human CHX10/VSX2 | 1:1,000 | X1179P | Exalpha Biologicals |
| Rabbit anti-Human CRX, Polyclonal | 1:1,000 | M231 | Takara Bio Inc. |
| Anti-MITF antibody (C5) | 1:1,000 | ab12039 | Abcam |
| Goat anti-Brn3a (C-20) antibody | 1:1,000 | sc-31984 | Santa Cruz Biotechnology Inc. |
| Mouse anti-Rhodopsin antibody, clone RET-P1 | 1:1,000 | MAB5316 | Sigma-Aldrich |
| Rabbit anti-Opsin antibody, Red/Green | 1:1,000 | AB5405 | Sigma-Aldrich |
| Mouse anti-CRALBP (B20) | 1:1,000 | ab15051 | Abcam |
| Mouse anti-PKC (MC5) | 1:200 | NB600-201 | Novus Biologicals |
| Goat anti-human NRL antibody | 1:200 | AF2945 | R&D systems |
| Mouse anti-RXRγ antibody (A-2) | 1:200 | sc-365252 | Santa Cruz Biotechnology Inc. |
| Rabbit anti-Recoverin antibody | 1:1,000 | 10073-1-AP | Proteintech Group Inc. |
| OPN1SW (N-20) | 1:1,000 | sc-14363 | Santa Cruz Biotechnology Inc. |
| Mouse anti-PROX1 Monoclonal antibody | 1:1,000 | 67438-1-Ig | Proteintech Group Inc. |
| Rabbit anti-Calretinin polyclonal antibody | 1:1,000 | 12278-1-AP | Proteintech Group Inc. |
| Donkey anti-Mouse IgG (H+L) Highly Cross-Adsorbed Secondary Antibody, Alexa Fluor 488 | 1:1,000 | A-21202 | Thermo Fisher Scientific Inc. |
| Donkey anti-Rabbit IgG (H+L) Highly Cross-Adsorbed Secondary Antibody, Alexa Fluor 488 | 1:1,000 | A-21206 | Thermo Fisher Scientific Inc. |
| Donkey anti-Goat IgG (H+L) Cross-Adsorbed Secondary Antibody, Alexa Fluor 546 | 1:1,000 | A-11056 | Thermo Fisher Scientific Inc. |
| Donkey anti-Rabbit IgG (H+L) Highly Cross-Adsorbed Secondary Antibody, Alexa Fluor 546 | 1:1,000 | A-10040 | Thermo Fisher Scientific Inc. |
| Donkey anti-Sheep IgG (H+L) Cross-Adsorbed Secondary Antibody, Alexa Fluor 546 | 1:1,000 | A-21098 | Thermo Fisher Scientific Inc. |
| Donkey anti-Mouse IgG (H+L) Highly Cross-Adsorbed Secondary Antibody, Alexa Fluor 647 | 1:1,000 | A-31571 | Thermo Fisher Scientific Inc. |
