# Supplementary material for: Rapid and efficient generation of mature retinal organoids derived from human pluripotent stem cells via optimized pharmacological modulation of Sonic hedgehog, activin A, and retinoic acid signal transduction
Source: PLoS One. 2024 Aug 9;19(8):e0308743. doi: 10.1371/journal.pone.0308743 (PMC11315325; doi:10.1371/journal.pone.0308743)
Supplement: S2 Table — (DOCX) [file pone.0308743.s002.docx]

**S2 Table. Primers for qPCR**

| Gene | Orientation | Sequence (5′–3′) |
| --- | --- | --- |
| *GAPDH* | Forward | GCACCGTCAAGGCTGAGAAC |
|  | Reverse | TGGTGAAGACGCCAGTGGA |
| *CRX* | Forward | CAGGAGTGGCTCAAGAGTCCAG |
|  | Reverse | CCCAGGGCACAATATTGTCTCA |
| *MITF* | Forward | AGGCATGAACACACATTCACGAG |
|  | Reverse | CAGGATCCATCAAGCCCAAGA |
| *RAX* | Forward | CGTCCCTAAGCGTGCTTTCA |
|  | Reverse | TTGCTCAGGACCGACAGACAGTA |
| *PAX6* | Forward | AGATGAGGCTCAAATGCGACTTC |
|  | Reverse | TCTCGGGCAAACACATCTGG |
