# Supplementary figures and images for: Rapid and efficient generation of mature retinal organoids derived from human pluripotent stem cells via optimized pharmacological modulation of Sonic hedgehog, activin A, and retinoic acid signal transduction
Source: PLoS One. 2024 Aug 9;19(8):e0308743. doi: 10.1371/journal.pone.0308743 (PMC11315325; doi:10.1371/journal.pone.0308743)

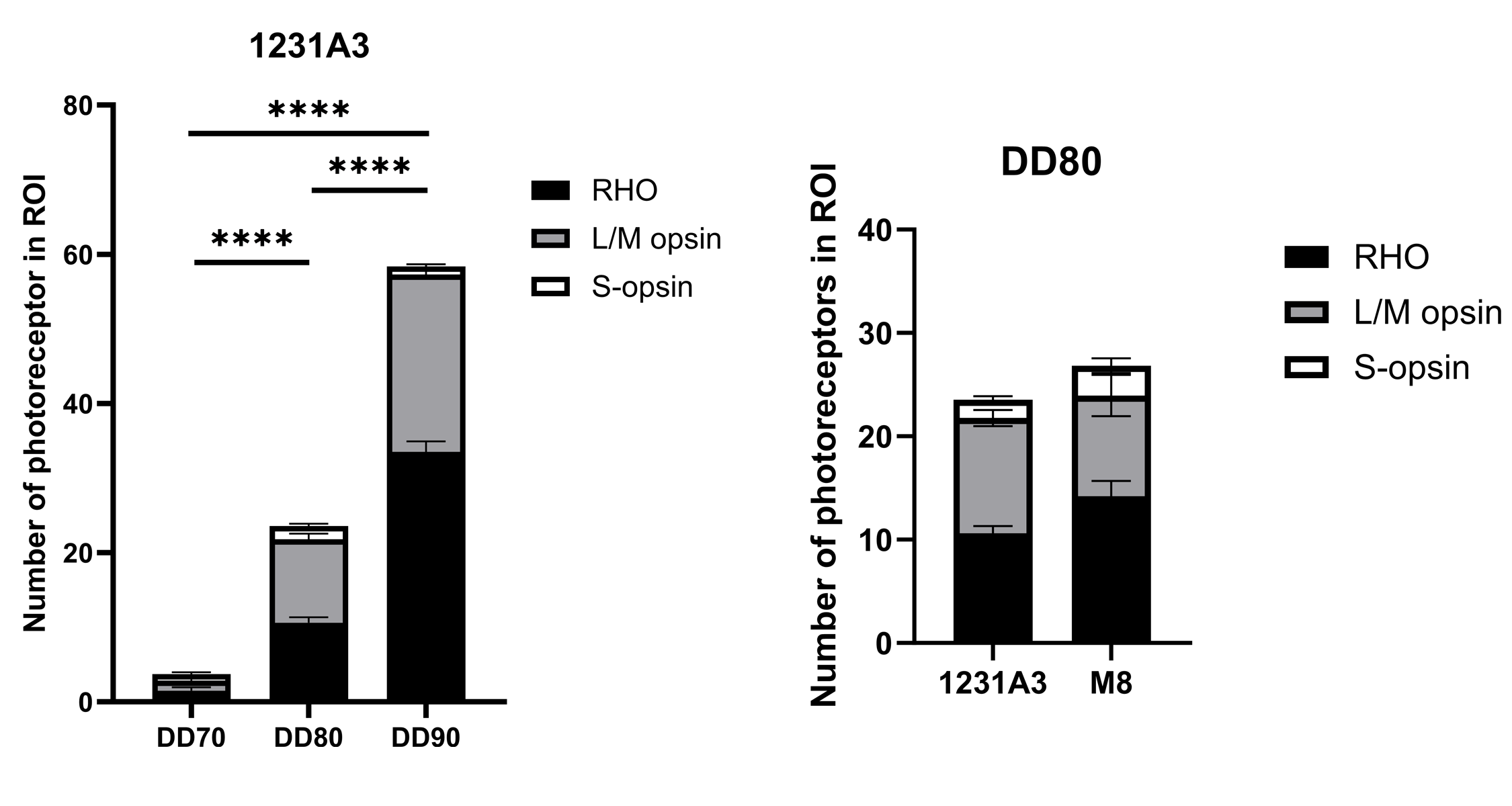

Supplement: S1 Fig — Retinal organoids differentiated from the human iPSC line 1231A3 by the present method were harvested at DD70, DD80, and DD90. Retinal organoids differentiated from the human iPSC line M8 by the present method were harvested at DD80. Retinal organoids were cryosectioned and immunostained with antibodies against photoreceptor markers rhodopsin, L/M opsin, and S opsin. A 300 μm-wide region of interest was randomly selected from 18 sections prepared from 18 retinal organoids, and fluorescent microscopic images were acquired using an FX3000 confocal laser scanning microscope (Olympus, Tokyo, Japan). Cell numbers were manually counted using FIJI-ImageJ software (National Institute of Health, Bethesda, USA). Data represent an arbitrarily chosen experiment from three independent experiments. Each bar represents the mean standard error of the photoreceptor numbers obtained from 18 individual sections of the 18 retinal organoids. ****P < 0.0001 by Tukey’s multiple comparison test. Statistical analyses were performed using GraphPad Prism version 10.1.0 for Windows (GraphPad Software, La Jolla, CA, USA). (TIF) [file pone.0308743.s003.tif]

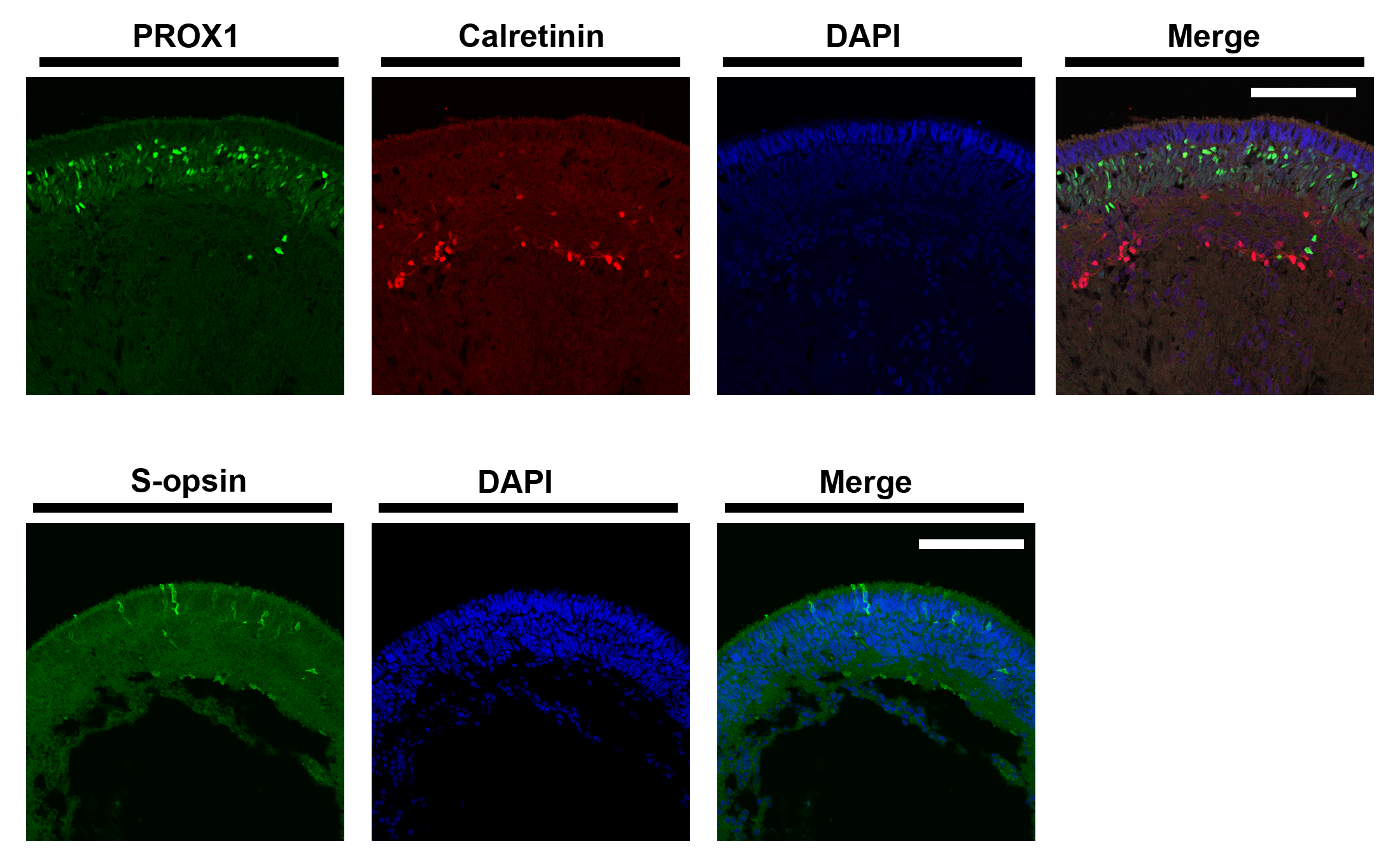

Supplement: S2 Fig — Retinal organoids differentiated from the human iPSC line 1231A3 by the present method were harvested at DD90. Retinal organoids were cryosectioned and immunostained using antibodies against various retinal cell marker proteins. PROX1 (upper, green): horizontal cells, calretinin (upper, red): amacrine cells, S opsin (lower, green): short-wave cone photoreceptors. The nuclei (blue) were stained with DAPI. Scale bar: 100 μm. The experiments were performed thrice with three different differentiation lots, in which six sections from six organoids were examined. Representative images were chosen arbitrarily. (TIF) [file pone.0308743.s004.tif]

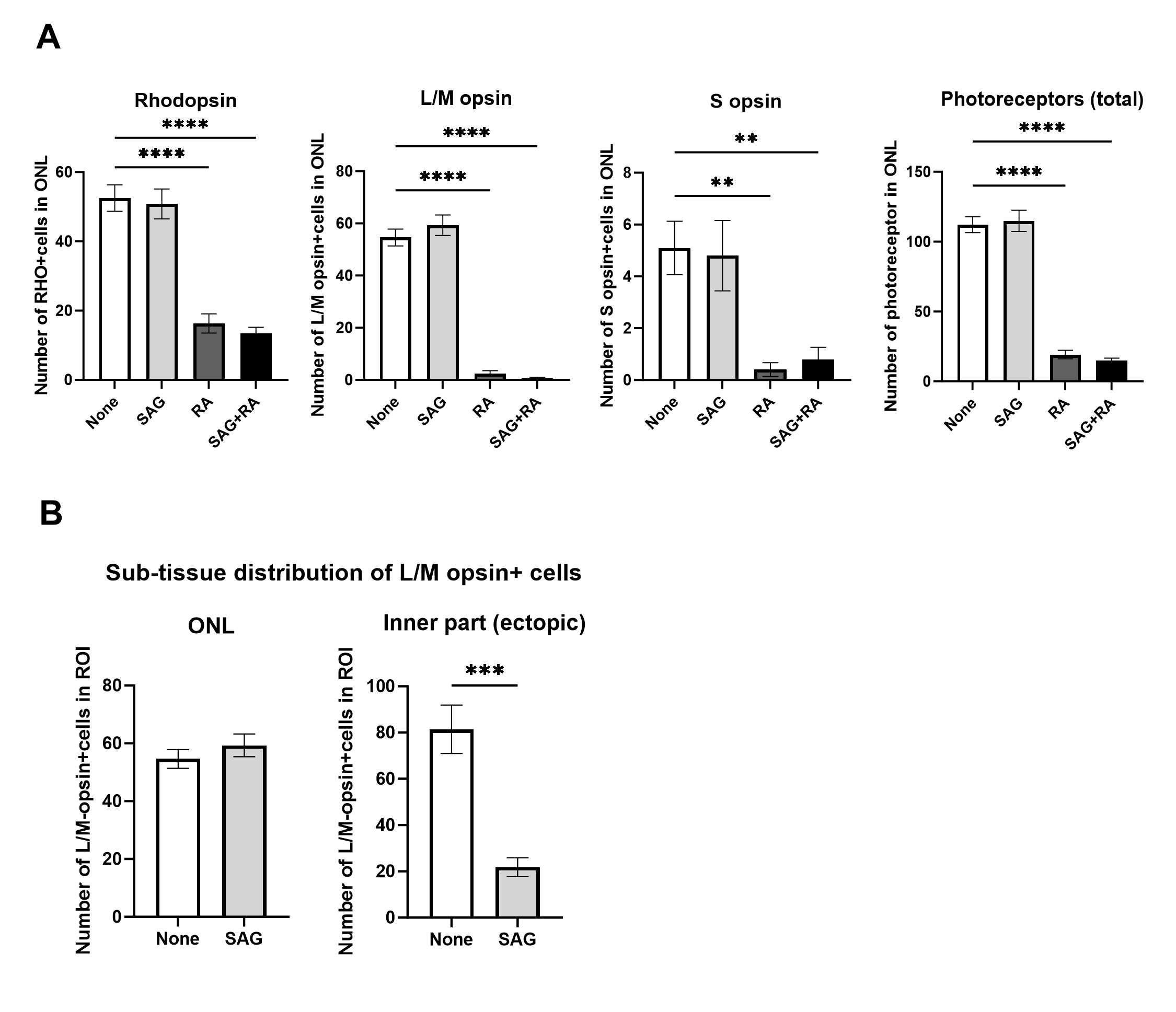

Supplement: S3 Fig — At DD90, retinal organoids were cryosectioned and immunostained with antibodies against the photoreceptor markers rhodopsin, L/M opsin, and S opsin. A 300 μm-wide region of interest was randomly selected from 10 sections prepared from 10 retinal organoids, and fluorescent microscopic images were acquired using an FX3000 confocal laser scanning microscope (Olympus, Tokyo, Japan). Cell numbers were manually counted using Fiji-ImageJ software (National Institute of Health, Bethesda, USA). Data represent an arbitrarily chosen experiment from three independent experiments. Each bar represents the mean standard error of the photoreceptor numbers obtained from 10 individual sections. (A) The effect of SAG and RA, either alone or in combination, on photoreceptor generation in ONL. **P < 0.01, ****P < 0.0001 by Dunnett’s multiple comparison test. (B) The effect of SAG on suppressing the ectopic expression of L/M opsin was analyzed by comparing the sub-tissue localization of L/M opsin-positive cells. ***P < 0.001 by Student’s t-test. Statistical analyses were performed using GraphPad Prism version 10.1.0 for Windows (GraphPad Software, La Jolla, CA, USA). (TIF) [file pone.0308743.s005.tif]

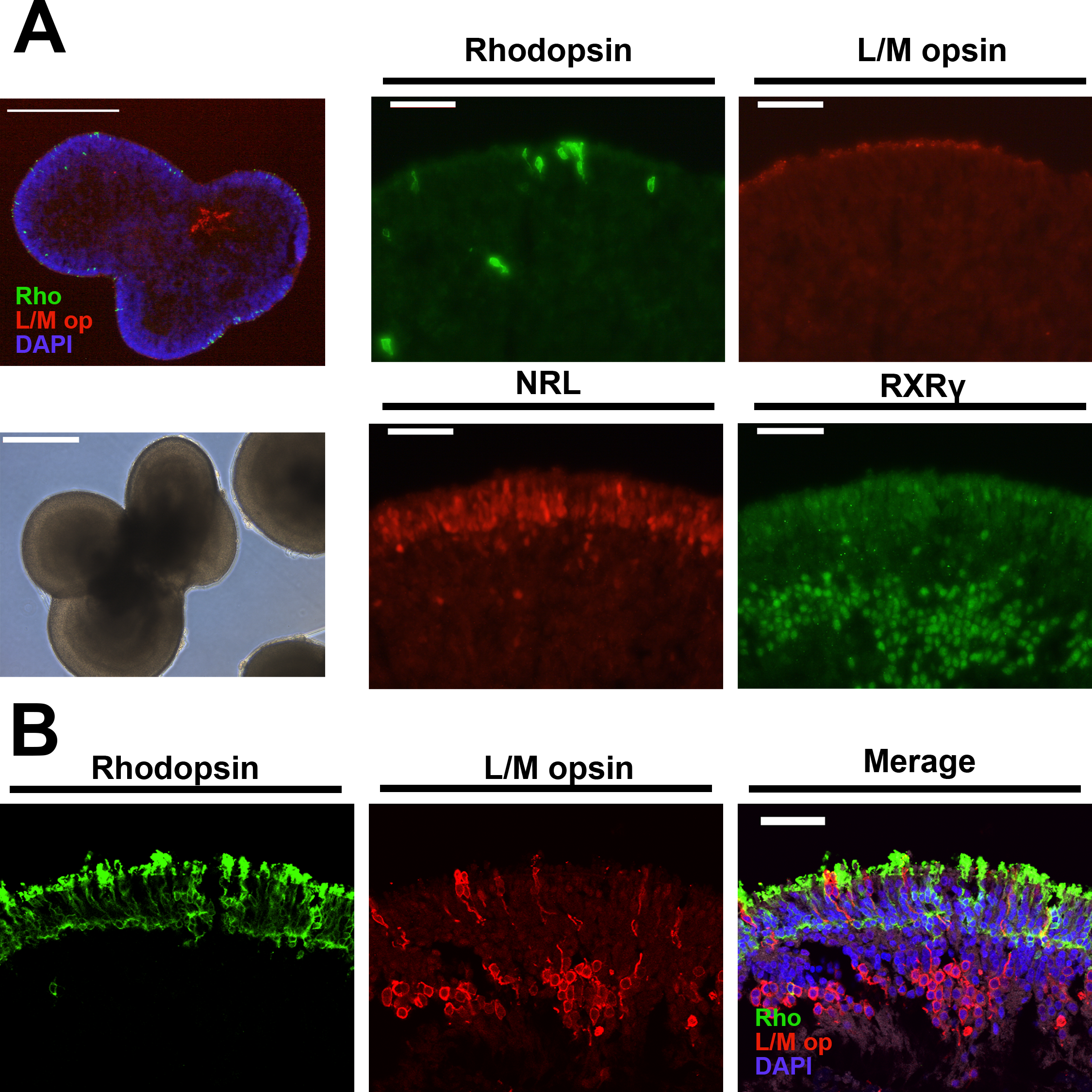

Supplement: S4 Fig — Retinal organoids were differentiated from human iPSC M8 according to the SFEBq method reported by Kuwahara et al. [20] and harvested at DD120 and DD222. Retinal organoids were cryosectioned and immunostained using antibodies against photoreceptor markers. The nuclei (blue) were stained with DAPI. (A) DD120 retinal organoids differentiated by the SFEBq method showed few rhodopsin-positive cells and no L/M opsin-positive cells, while immature rod and cone photoreceptor markers, NRL and RXRγ, respectively, were expressed. Scale bars: 500 μm (left) and 100 μm (middle and right). (B) DD222 retinal organoids differentiated by the SFEBq method showed rhodopsin (green) and L/M opsin (red) expression. Scale bar: 50 μm. The experiments were performed thrice with three different differentiation lots, in which six sections from six organoids were examined. Representative images were chosen arbitrarily. (TIF) [file pone.0308743.s006.tif]
